# Palmitoyl‐Protein Thioesterase 1 (PPT1) Protein, Linked to Neuronal Ceroid Lipofuscinosis 1, Is a Major Constituent of Ageing‐Related Human Neuronal Lipofuscin

**DOI:** 10.1111/nan.70043

**Published:** 2025-09-24

**Authors:** Max Anstötz, Sarah Tschirner, Caroline May, Steffen Kösters, Christine Martin, Svenja Caspers, Eleonora Aronica, Hans Jürgen Bidmon, Katrin Marcus, Carsten Korth

**Affiliations:** ^1^ Institute of Anatomy II, Medical Faculty Heinrich Heine University Düsseldorf Düsseldorf Germany; ^2^ Department of Neuropathology Heinrich Heine University Düsseldorf Düsseldorf Germany; ^3^ Center for Protein Diagnostics (PRODI), Medical Proteome Analysis Ruhr‐University Bochum Bochum Germany; ^4^ Medical Faculty, Medizinisches Proteom‐Center Ruhr‐University Bochum Bochum Germany; ^5^ Institute for Anatomy I, Medical Faculty and University Hospital Düsseldorf Heinrich Heine University Düsseldorf Düsseldorf Germany; ^6^ Institute of Neurosciences and Medicine, Structural and Functional Organisation of the Brain (INM‐1) Forschungszentrum Jülich Jülich Germany; ^7^ Department of (Neuro)Pathology, Amsterdam UMC University of Amsterdam, Amsterdam Neuroscience Amsterdam the Netherlands; ^8^ C. & O. Vogt Institute of Brain Research Heinrich Heine University Düsseldorf Düsseldorf Germany

**Keywords:** brain ageing, ceroid, lipofuscin, lysosome, neuronal ceroid lipofuscinosis, Palmitoyl‐Protein Thioesterase 1

## Abstract

- Proteomics of laser‐dissected lipofuscin from aged, healthy brains reveals Palmitoyl‐Protein Thioesterase 1 (PPT1) and other CLN proteins as constituents.
- PPT1 is increasingly sequestered to lipofuscin during ageing.
- Protein sequestering into lipofuscin may contribute to physiological neuronal ageing.

The nature of age‐associated neuronal brain lipofuscin remains unknown. To date, the term ‘lipofuscin’ is somewhat ambiguous. It was coined by Hueck [1] over a century ago and broadly refers to cellular material deposited in lysosomes, consisting of proteins, lipids and inorganic material in various proportions and thus remains poorly defined in precise molecular terms. Lipofuscin is known to be present in postmitotic tissues associated with lysosomes and to accumulate in an age‐associated manner as autofluorescent material [2]. It has been hypothesised that lysosomes surrounding lipofuscin are neither able to degrade nor to secrete this material, which therefore accumulates in a strictly age‐dependent manner [3].

Autofluorescent brain lipofuscin has long been regarded as mere cellular debris accumulating over time, and research on its molecular composition is sparse. A key question is whether lipofuscin accumulation reflects aberrant proteostasis during physiological ageing, and whether it contributes to age‐associated brain changes by impairing neuronal cellular homeostasis or facilitates age‐associated brain diseases. Some recent findings indeed indicate that lipofuscin could play a role in neurodegenerative diseases by impairing lysosomal degradation of aggregated proteins [3].

Two independent facts support the disease‐relevant function of lipofuscin: (1) the existence of a family of inherited neuronal ceroid lipofuscinoses (NCL) of heterogeneous genetic origin [4] and (2) neurotoxic lipofuscin accumulating in retinal pigment epithelium (RPE) in age‐associated macular degeneration, the most frequent cause of blindness in the Western hemisphere [5, 6].

NCLs are childhood neurodegenerative diseases with variable ages of onset and penetrance, featuring accumulating autofluorescent material (termed ceroid) in neurons, progressive cognitive impairment of variable degrees, epilepsy and other severe neurological symptoms. Interestingly, the products of different ceroid lipofuscinosis neuronal (CLN) genes, though seemingly unrelated in function, interact within protein networks and overlap with proteins involved in neurodegenerative diseases [4].

In contrast to the molecular analysis of neuronal brain lipofuscin, retinal lipofuscin in RPE is better characterised in terms of its molecular constituents [7]. The neurotoxic properties of RPE lipofuscin have been attributed to the small organic molecule N‐retinylidene‐N‐retinylethanolamine (A2E [5]).

We previously analysed human and rat age‐associated brain lipofuscin by proteomics, subsequent to a biochemical purification of frozen brains and reported overlapping protein content between aged human and rat lipofuscin [8]. However, the major caveat of this biochemical isolation protocol is that after lysis and during homogenization, protein constituents of different cell organelles or cytosol can mistakenly physically adhere to macromolecular lipofuscin and thus contaminate its true composition. An alternative approach to minimise contamination is to dissect lipofuscin directly from brain neurons and subsequently subject it to a proteomic analysis.

Here we analysed human lipofuscin from the anterior hippocampus of an aged individual (86 years) by laser microdissection (LMD), where single lipofuscin particles were isolated and analysed by electrospray mass spectrometry followed by peptide fingerprinting (see Section S1). We dissected approximately 4200 intracellular spots (Figures S1 and S2). As control material, against which the dissected lipofuscin content was compared, we dissected intracellular material immediately adjacent to lipofuscin from the same cells but non‐autofluorescent (see Figures S1 and S2). After liquid chromatography‐coupled tandem mass spectrometry (LC–MS/MS) of the material, we performed peptide fingerprinting and quantitative comparison of lipofuscin versus control using label‐free quantitation (LFQ). Even though a high number in LFQ is dependent on many factors unrelated to abundance, we observed a strong twofold to tenfold increase of lipofuscin LFQ in the top 10 hits compared to all other hits (Table S1 for complete list).

The highest LFQ scores were counted for proteins calmodulin, (pro)saposin, nephronectin, and ATP subunit D. Since for calmodulin, a significant portion was also identified in the control tissue, we hesitate to assign equal relevance to calmodulin as compared to the other identified top lipofuscin proteins, where presence was exclusive to lipofuscin‐dissected tissue. Surprisingly, among the top hits was Palmitoyl‐Protein Thioesterase 1 (PPT1), a protein that, when deficient or mutant, causes CLN1, a specific NCL, suggesting potential links between sporadic, autofluorescent ageing‐associated lipofuscin and genetic forms of autofluorescent ceroid. Therefore, we decided to specifically validate the presence of PPT1 in human aged versus juvenile neuronal, hippocampal lipofuscin as a representative example.

We confirmed the presence of PPT1 in lipofuscin in the hippocampus of several different‐aged human brain samples and demonstrated its co‐localisation with lipofuscin (Figures 1A–C and S3). To assess the differential cellular co‐localisation of PPT1 from aged and juvenile individuals, as well as to explore age‐dependent cellular re‐distribution of PPT1 in neurons of human hippocampus, we performed high‐resolution light microscopy with an anti‐PPT1 immunolabelling in juvenile (*n* = 3) and aged brains (*n* = 5; Figures 1D–G and S3B,C). Whereas in the juvenile brain, PPT1 was distributed in lipofuscin and non‐lipofuscin compartments, in aged brains, the association of PPT1 to lipofuscin was at least fivefold higher (Figure 1G). These findings demonstrate that PPT1 associates with lipofuscin in an age‐dependent manner such that the relative amount of freely distributed, intracellular PPT1 decreases.

**FIGURE 1:**
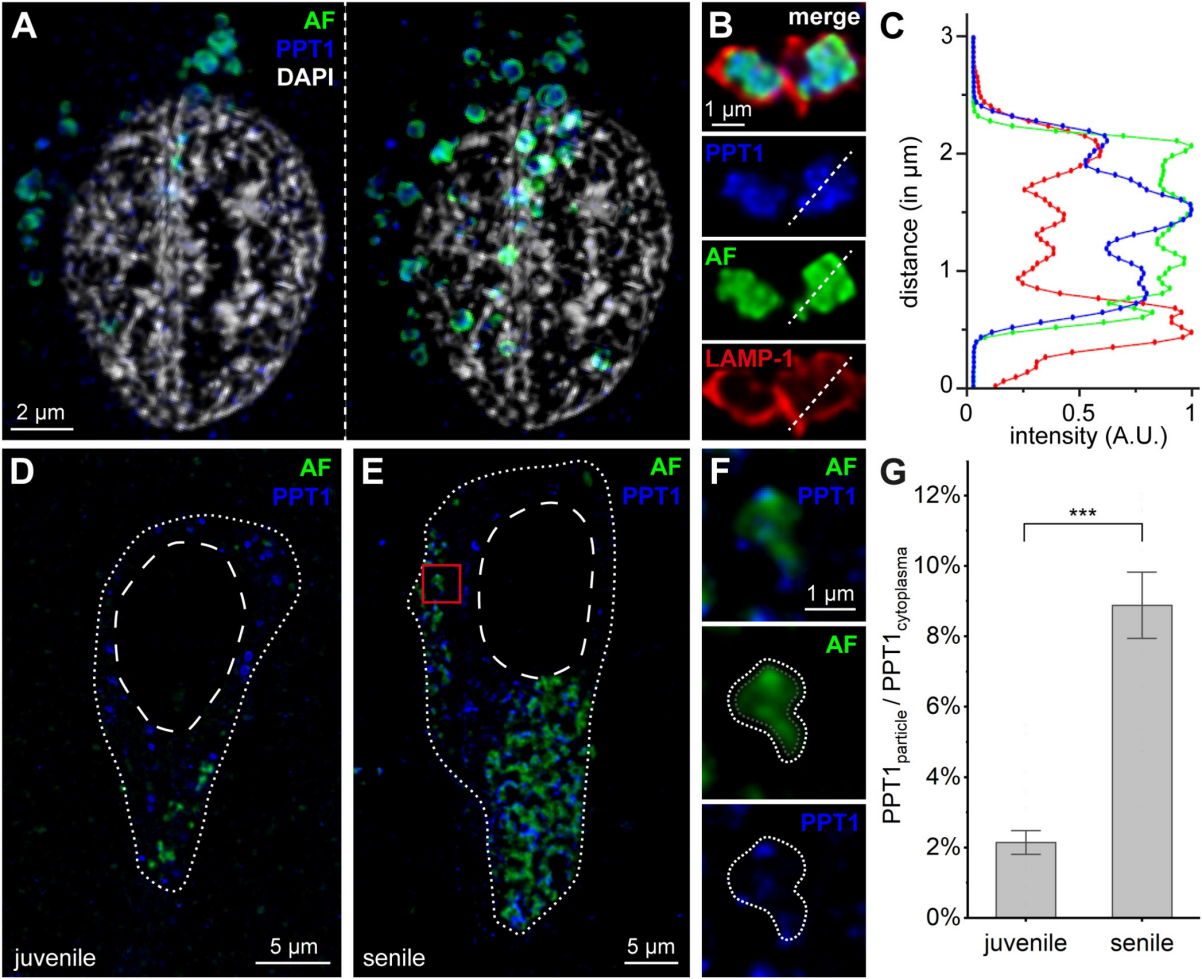
(A) Representative, deconvoluted confocal microscopy image of an immunohistochemical staining of a hippocampal CA1 pyramidal cell soma in a senile human. Lipofuscin (autofluorescence: AF, green), PPT1 (blue) and DAPI (white) are shown. Left panel: single thin optical section. Right panel: Superimposed stack of multiple *Z* sections. (B) Representative high‐magnification image of two lipofuscin particles co‐localised with the lysosomal marker LAMP‐1 (red) and PPT1 (blue). Note the surrounding LAMP‐1 immunofluorescence (red) and the clustered appearance of PPT1 (blue) within a lipofuscin particle (green). Upper panel: merged channels, second panel PPT1, third panel autofluorescence, lower panel LAMP‐1. A dashed line indicating the line used for subsequent analysis with a line‐intensity profile of separated fluorescence channels. (C) Line‐intensity profile of the fluorescence channels shown in B. The profiles reveal co‐localisation of the signals, yet not complete overlap, indicating that the lipofuscin particle is surrounded by a lysosomal membrane (LAMP‐1, red), with PPT1 (blue) appearing as clustered signals within the particle. (D) Representative, deconvoluted confocal microscopy image of a representative CA1 pyramidal cell in a juvenile hippocampus, showing lipofuscin autofluorescent particles (AF, green) and PPT1 immunofluorescence (blue). The dotted line outlines the soma, while the dashed line outlines the nucleus. (E) Same as in (D), but for a CA1 pyramidal neuron in a senile hippocampus. Note the higher density of autofluorescent particles and the co‐localised PPT1 immunofluorescence. (F) High‐magnification view of an autofluorescent lipofuscin particle outlined in (D) (red box). The upper panel shows the superimposed image, the middle panel shows the isolated autofluorescent lipofuscin particle, and the lower panel shows the PPT1 immunofluorescence. The dotted line indicates the outlined particle area plus a 100‐nm margin, used for subsequent analysis of PPT1 immunofluorescence co‐localised with lipofuscin particles. (G) Bar plot showing the mean ± standard error of the area fraction of PPT1 co‐localised with lipofuscin particles relative to total cytoplasmic PPT1. Statistical analysis was performed using the Mann–Whitney U test (*n* = 10 cells per specimen, *n* = 3 juvenile specimens/*n* = 5 aged specimens, ***p < 0.001).

PPT1 has been detected in various intracellular compartments and the cytosol, where it catalyses the depalmitoylation of fatty acids for the regulation of protein trafficking, lysosomal degradation or synaptic maintenance [9, 10]. PPT1 has been reported to form small aggregate‐like structures in cells [9], and it is tempting to speculate that these aggregates may be sequestered into lipofuscin. As a consequence, the sequestering of insoluble PPT1 could lead to a functional loss of soluble PPT1 that cannot be compensated in the context of the ageing brain. It is conceivable that this functional loss of PPT1 by sequestration and that of other lipofuscin‐associated proteins could lead to subtle functional deficits of ageing neurons.

At much lower LFQ levels, other proteins encoded by CLN genes were also detected in lipofuscin (see Table S1): tripeptidyl peptidase 1 (CLN2), DNaJ heat shock protein family member C5 (CLN4), cathepsin D (CLN10), progranulin (CLN11), ATPase 13A2 (CLN12) and cathepsin F (CLN13). Cathepsin D, however, was also identified in significant amounts in control tissue.

While the omnipresence of lipofuscin in the aged brain does not per se predispose one to neurodegenerative disease, ageing does [11]. Lipofuscin may be one of many cellular factors that contribute to this risk. Notably, among the other top hits in the protein constituents of lipofuscin (Table S1), the presence of saposin is remarkable, as increased levels have been correlated with increased pathology in Alzheimer's disease patients [12], supporting the view that lipofuscin, as an ageing‐dependent factor, may contribute to sporadic ad.

In summary, we present an in situ protein analysis of age‐associated human brain lipofuscin that, among others, reveals PPT1 protein as one major constituent even though PPT1 physiologically does not have an exclusive lysosomal presence. It remains to be demonstrated whether PPT1's age‐associated sequestration to lipofuscin leads to its functional impairments in affected neurons and whether such a functional impairment would bear any similarity to functional impairments in CLN diseases.

## Author Contributions

CK, MA, and ST conceived the study. MA, ST, CaM, SK, and ChM performed experiments. MA, CaM, SC, EA, HJB, KM and CK provided critical materials. All authors contributed to data analysis. CK, MA, ST, and HJB wrote the manuscript. All authors contributed comments to the discussion of the results and the manuscript.

## Ethics Statement

All post‐mortem brains were obtained after written, informed consent from donors or authorised relatives. All procedures were conducted in accordance with the guidelines for good laboratory practice of the European Commission, in accordance with the Declaration of Helsinki and local research codes. Relevant positive ethics votes were obtained prior to all procedures from the Ethics Committee of the Medical Faculty of the Heinrich Heine University of Düsseldorf (#4863 and #2023‐2632) for brains from senile individuals or the Ethics Committee from Amsterdam University Medical Center (#W21_295) for brains from juvenile individuals.

## Conflicts of Interest

The authors declare no conflicts of interest.

## Supporting information


**Figure S1:** Selection of lipofuscin spots for LMD in human posterior hippocampal tissue. Lipofuscin areas were selected and marked for subsequent excision based on their autofluorescent properties as depicted in A and B. Selected spots are highlighted by green arrows. Selection was performed in green fluorescence mode, while the bright field mode was used for subsequent excision. C and D show the corresponding bright field images where lipofuscin is visible as yellow‐brownish pigment. Scale bars: 75 μm.
**Figure S2:** Sampling of a lipofuscin‐free adjacent tissue control. On average, two control areas (red labels and arrows) were selected next to every fifth lipofuscin spot (green labels and arrows). As for lipofuscin itself, the selection was conducted in green fluorescence mode when lipofuscin was already excised (A and B). In C and D, brain tissue is displayed in bright field mode after lipofuscin and control area collection. Scale bars: 75 μm.
**Figure S3:** A) Epi‐fluorescence and brightfield microscopy images of a senile hippocampal section. The merged image combines inverted and brightfield oblique illumination (Obl. Illum., red), DAPI nuclear staining (blue), and autofluorescence (green). Labelled regions include the dentate gyrus (DG), Cornu Ammonis 3 (CA3), and Cornu Ammonis 1 (CA1). Scale bar: 1 mm. B) Deconvoluted confocal microscopy images of an immunohistochemical staining of hippocampal CA1 pyramidal neurons from five individual senile human hippocampal sections. Note the varying density of lipofuscin (AF, green) and PPT1 (blue). Scale bar: 5 μm. Insets below show high‐power magnification of lipofuscin granules adjacent to PPT1. Scale: 4 μm (width of inset panel). C) Same as in B, but for neurons in juvenile human hippocampal sections. Scale is identical to B.

**Data S1:** Supplementary Table 1.

## Data Availability

The data that support the findings of this study are available on request from the corresponding author. The data are not publicly available due to privacy or ethical restrictions.
