# Supplementary material for: Palmitoyl‐Protein Thioesterase 1 (PPT1) Protein, Linked to Neuronal Ceroid Lipofuscinosis 1, Is a Major Constituent of Ageing‐Related Human Neuronal Lipofuscin
Source: Neuropathol Appl Neurobiol. 2025 Sep 24;51(5):e70043. doi: 10.1111/nan.70043 (PMC12459315; doi:10.1111/nan.70043)
Supplement: Supplementary file 1 — Figure S1: Selection of lipofuscin spots for LMD in human posterior hippocampal tissue. Lipofuscin areas were selected and marked for subsequent excision based on their autofluorescent properties as depicted in A and B. Selected spots are highlighted by green arrows. Selection was performed in green fluorescence mode, while the bright field mode was used for subsequent excision. C and D show the corresponding bright field images where lipofuscin is visible as yellow‐brownish pigment. Scale bars: 75 μm. Figure S2: Sampling of a lipofuscin‐free adjacent tissue control. On average, two control areas (red labels and arrows) were selected next to every fifth lipofuscin spot (green labels and arrows). As for lipofuscin itself, the selection was conducted in green fluorescence mode when lipofuscin was already excised (A and B). In C and D, brain tissue is displayed in bright field mode after lipofuscin and control area collection. Scale bars: 75 μm. Figure S3: A) Epi‐fluorescence and brightfield microscopy images of a senile hippocampal section. The merged image combines inverted and brightfield oblique illumination (Obl. Illum., red), DAPI nuclear staining (blue), and autofluorescence (green). Labelled regions include the dentate gyrus (DG), Cornu Ammonis 3 (CA3), and Cornu Ammonis 1 (CA1). Scale bar: 1 mm. B) Deconvoluted confocal microscopy images of an immunohistochemical staining of hippocampal CA1 pyramidal neurons from five individual senile human hippocampal sections. Note the varying density of lipofuscin (AF, green) and PPT1 (blue). Scale bar: 5 μm. Insets below show high‐power magnification of lipofuscin granules adjacent to PPT1. Scale: 4 μm (width of inset panel). C) Same as in B, but for neurons in juvenile human hippocampal sections. Scale is identical to B. [file NAN-51-e70043-s001.pdf]

Palmitoyl-Protein Thioesterase 1 (PPT1) protein, linked to neuronal ceroid lipofuscinosis 1, is a major constituent of ageing-related human neuronal lipofuscin

Max Anstötz[1+*], Sarah Tschirner[2+], Caroline May[3], Steffen Kösters[3], Christine Martin[1], Svenja Caspers[4, 6], Eleonora Aronica[5], Hans Jürgen Bidmon[6,7], Katrin Marcus[3], Carsten Korth[2*]

Supplementary Methods and Figures

Supplementary Methods

Human brain tissue for lipofuscin purification and staining

Lipofuscin was isolated from cryopreserved *post-mortem* posterior hippocampal tissue from a female 85-year-old male body donor. The donor was without any diagnosed neurological or neurodegenerative disorder and had given written informed consent for the removal of organs and tissues and their use for research purposes. For validation, right posterior hippocampus from an 84-year-old female (senile #1), 85-year-old male (senile #2), 81-year-old female (senile #3), 88-year-old female (senile #4) and a 83-year-old (senile #5) female were used (causes of death: heart insufficiency, renal insufficiency, hypertonia, cachexia, an respiratory insufficiency was diagnosed, respectively). Relevant Ethics votes #4863 and #2023-2632 had been obtained from the Ethics Committee oft he Medical Faculty of the Heinrich Heine University of Düsseldorf.

Juvenile brains samples (hippocampus) were obtained from the archives of the Department of Neuropathology of the Amsterdam UMC (Amsterdam, the Netherlands) including specimens from a 12-year-old male (juvenile #1), a 13-year-old female (juvenile #3), and a 17-year-old (juvenile #2) female (the causes of death were cardiomyopathy, cardiac arrhythmia, and pneumonia, respectively), after written, informed consent from authorised relatives. All procedures received prior approval by the local ethics committee (#W21_295), and were conducted in accordance with the guidelines for good laboratory practice of the European Commission and in accordance with the Declaration of Helsinki and the Amsterdam UMC Research Code. For control samples in these experiments, careful analysis and evaluation of clinical data were used to include samples that displayed normal cortical structure and no significant brain pathology.

Cryosectioning

With a CryoStar NX50 cryostat (Thermo Scientific, Germany), frozen posterior hippocampal tissue was sectioned into slices of 10 µm thickness and transferred onto 1.0 PEN membrane glass slides for laser microdissection (LMD) (Carl Zeiss Microscopy GmbH, Göttingen, Germany) with one tissue slice per slide similar to what described before [1]. Slides were then placed in plastic containers, laminated to prevent hydration and stored at -80 °C until use for LMD.

Lipofuscin purification by laser microdissection (LMD)

LMD was performed using a ZEISS PALM Microbeam Laser Microdissection System (Carl Zeiss Microscopy GmbH) that was operated with the software PALMRobo 4.6 pro (Carl Zeiss Microscopy GmbH) similar as described [1]. First, slices were dehydrated by 2 min incubation in pre-cooled 70% ethanol followed by short dipping into pre-cooled absolute ethanol for two or three times. Slices not directly needed were kept frozen. One dehydrated and air-dried slice was then placed under the LMD microscope and an overview scan at 50-fold magnification was made for subsequent navigation and tissue spot selection. Lipofuscin areas were selected and marked based on lipofuscin autofluorescence at 400-fold magnification in green fluorescence mode using an exposure time of 0.5-1 sec. Lipofuscin spots were randomly checked for fluorescence also in red and blue channel as well as for yellow-brownish coloring in bright field mode. Collection was performed at the same magnification in bright field mode, and spots were excised and catapulted into 50 µL of distilled water that was added to the lid of a non-adhesive microtube (MicroTube 500, Carl Zeiss Microscopy GmbH) placed above the tissue slice. On average 120 lipofuscin spots were marked and collected in one round until a

total number of roughly 4200 spots. After each round, lipofuscin-free control spots were selected adjacent to the lipofuscin areas with two controls next to every fifth lipofuscin spot on average. Control area selection and sampling was performed as described for lipofuscin except for an elevated exposure time of 1-2 sec in order to avoid the excision of small lipofuscin deposits with only weak fluorescence signal. Control tissue was collected in a separate tube. The tissue area excised in total was 1.32 x 10^6 μm^2 for lipofuscin and 1.092 x 10^6 μm^2 for the control. Samples were kept at -80 °C until further processing. Lipofuscin and control spot selection is illustrated in Supplementary Fig. 1. Mass spectrometric analysis as well as data analysis using MaxQuant was performed as described [2].

Immunohistochemistry of free floating hippocampal sections

Hippocampi were dissected from the medial temporal lobe and fixed in 4% formaldehyde for at least 72 hours at 4°C. After fixation, the tissue was transferred to 0.01 M phosphate-buffered saline (PBS). Sections were then cut into 50 µm-thick slices using a Leica VT1000 vibratome and collected free-floating in PBS.

For fluorescence immunohistochemistry, the slices were preincubated for 1 hour at room temperature (RT) in a blocking solution containing 5% normal goat serum (NGS), 1% bovine serum albumin (BSA), and 0.5% Triton X-100 in PBS. After blocking, the slices were incubated overnight at 4°C with primary antibodies: rabbit anti-PPT1 (1:500, Sigma-Aldrich, Cat# HPA021546) and mouse anti-LAMP1 (1:500, DSHB, Cat# H4A3).

Following primary antibody incubation, the slices were washed three times for 15 minutes each with fresh PBS. They were then incubated at RT for 1 hour in a solution containing 5% NGS and 1% BSA in PBS, along with the secondary antibodies: Alexa Fluor Plus 405 goat anti-mouse IgG (1:500, ThermoFisher, Cat# A48255) and Alexa Fluor Plus 647 (1:500, ThermoFisher, Cat# A32733). Finally, the sections were washed again three times for 15

minutes each with PBS, then mounted and cover-slipped using Fluoromount-G mounting medium (ThermoFisher, Cat# 00-4958-02).

Immunohistochemistry of paraffin sections

Paraffin sections of juvenile and aged hippocampi were deparaffinized using Rotihistol (Carl Roth, Cat# 6640.1) for 2 × 5 minutes, followed by rehydration at room temperature (RT) with 100% ethanol (2 × 5 minutes), and 90%, 80%, and 70% ethanol (1 minute each). The sections were then transferred to 0.01 M PBS for 5 minutes. Subsequent immunohistochemistry was performed as described above for free-floating sections using the anti-PPT1 antibody. Finally, the sections were cover-slipped using Fluoromount-G mounting medium.

Imaging and analysis of PPT-1 distribution

Sections were imaged using a Leica SP8 confocal microscope equipped with a 100× NA 1.4 oil-immersion lens (Leica HC PL APO CS2). Image deconvolution was performed using the Leica Lightning deconvolution plugin, with a plugin-calculated resolution of 35 nm/pixel. For the analysis of PPT1 distribution adjacent to autofluorescent lipofuscin particles, a custom ImageJ plugin was used. First, a threshold-based selection of lipofuscin particles in the cytosol was created and subsequently expanded by a margin of 100 nm. The intensity of the PPT1 immunofluorescence channel within this selection was then divided by the total PPT1 immunofluorescence intensity.

Supplementary Figures

Supplementary Figure 1

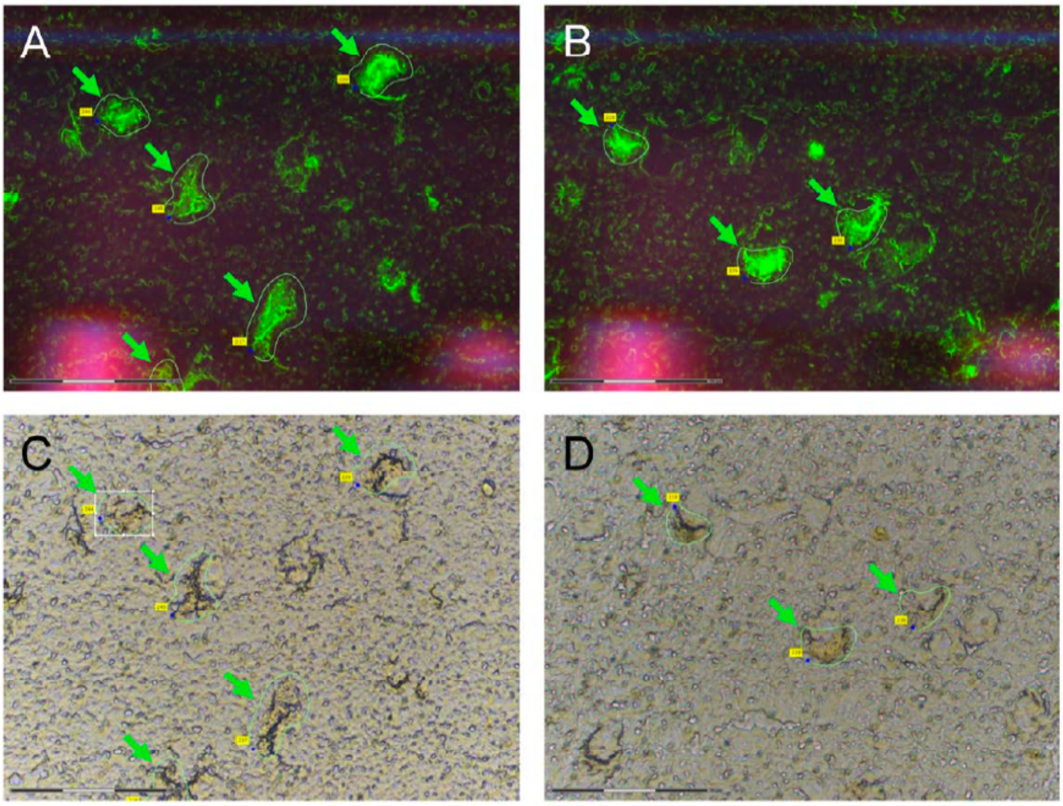

Supplementary Figure S1: Selection of lipofuscin spots for LMD in human posterior hippocampal tissue. Lipofuscin areas were selected and marked for subsequent excision based on their autofluorescent properties as depicted in A and B. Selected spots are highlighted by green arrows. Selection was performed in green fluorescence mode, while the bright field mode was used for subsequent excision. C and D show the corresponding bright field images where lipofuscin is visible as yellow-brownish pigment. Scale bars: 75 µm.

Supplementary Figure S2

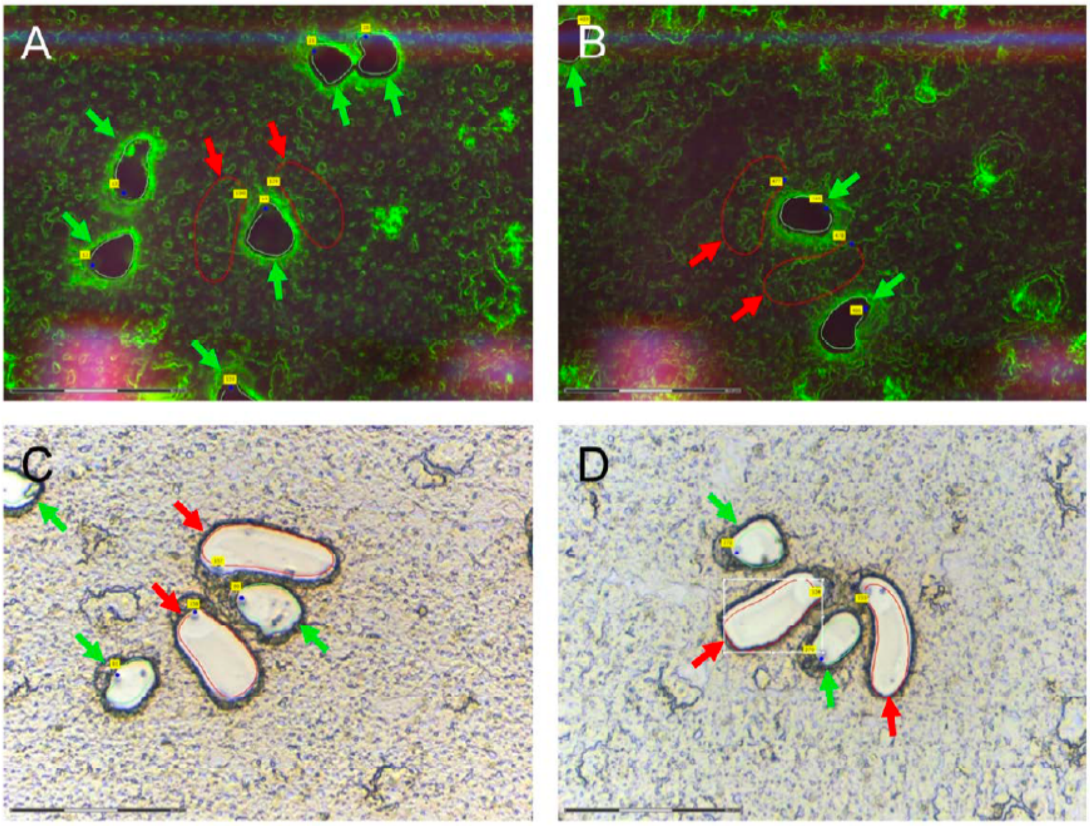

Supplementary Figure S2: Sampling of a lipofuscin-free adjacent tissue control.

On average, two control areas (red labels and arrows) were selected next to every fifth lipofuscin spot (green labels and arrows). As for lipofuscin itself, the selection was conducted in green fluorescence mode when lipofuscin was already excised (A and B). In C and D, brain tissue is displayed in bright field mode after lipofuscin and control area collection. Scale bars: 75 µm.

Supplementary Figure 3

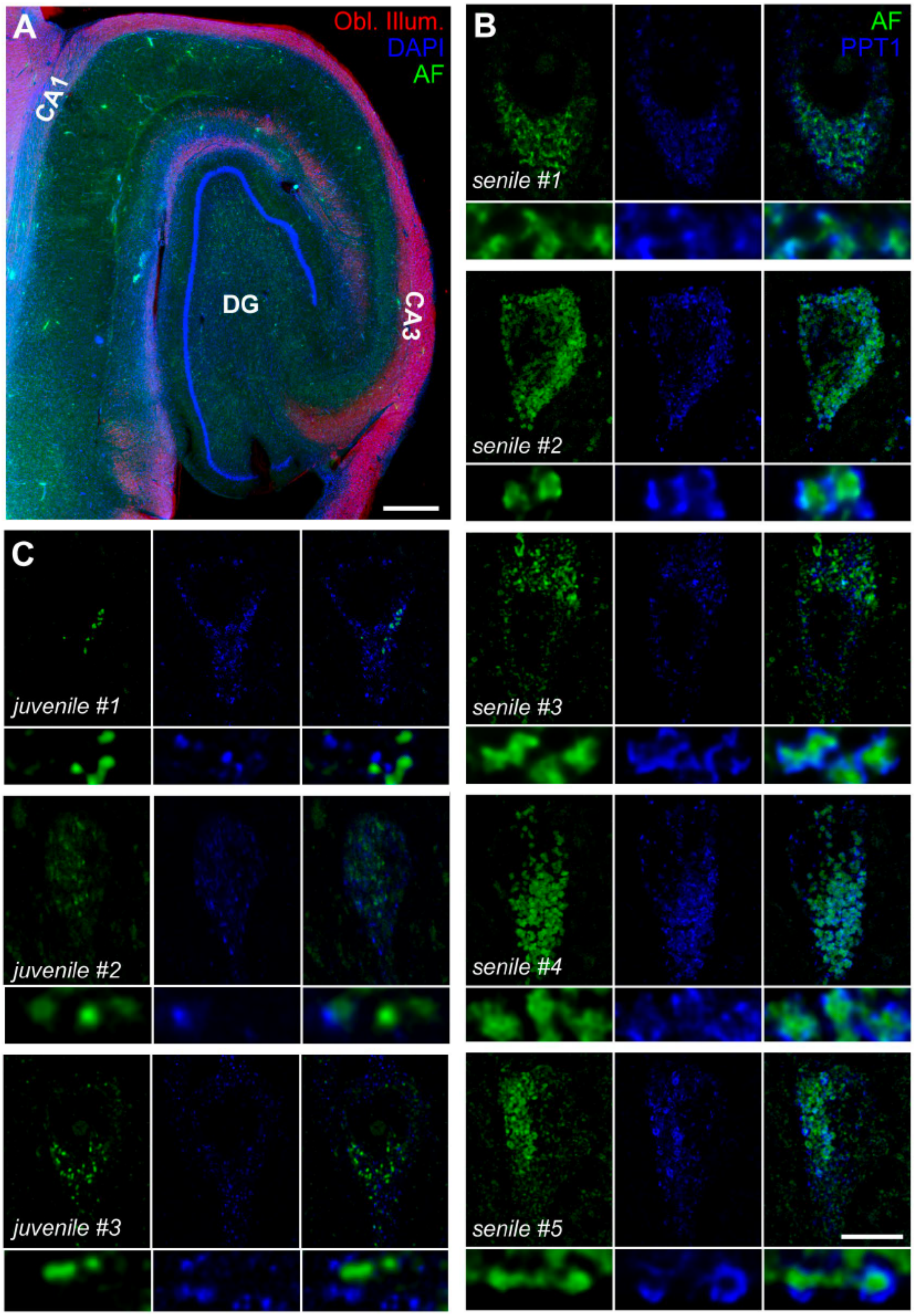

Supplementary Figure 3: A) Epi-fluorescence and brightfield microscopy images of a senile hippocampal section. The merged image combines inverted and brightfield oblique illumination (Obl. Illum., red), DAPI nuclear staining (blue), and autofluorescence (green). Labeled regions include the dentate gyrus (DG), Cornu Ammonis 3 (CA3), and Cornu Ammonis 1 (CA1). Scale bar: 1 mm.

B) Deconvoluted confocal microscopy images of an immunohistochemical staining of hippocampal CA1 pyramidal neurons from five individual senile human hippocampal sections. Note the varying density of lipofuscin (AF, green) and PPT1 (blue). Scale bar: 5 µm. Insets below show high-power magnification of lipofuscin granules adjacent to PPT1. Scale: 4 µm (width of inset panel).

C) Same as in B, but for neurons in juvenile human hippocampal sections. Scale is identical to B.

References

1 Plum S, Steinbach S, Attems J, Keers S, Riederer P, Gerlach M, May C, Marcus K. Proteomic characterization of neuromelanin granules isolated from human substantia nigra by laser-microdissection. Sci Rep 2016; 6: 37139

2 Plum S, Eggers B, Helling S, Stepath M, Theiss C, Leite REP, Molina M, Grinberg LT, Riederer P, Gerlach M, May C, Marcus K. Proteomic Characterization of Synaptosomes from Human Substantia Nigra Indicates Altered Mitochondrial Translation in Parkinson's Disease. Cells 2020; 9:
